# Challenges and Solutions for Musculoskeletal Disorders in Athletes

**DOI:** 10.3390/medicina58010080

**Published:** 2022-01-05

**Authors:** Giovanni Iolascon, Umberto Tarantino, Antimo Moretti

**Affiliations:** 1 Department of Medical and Surgical Specialties and Dentistry, University of Campania “Luigi Vanvitelli”, 80100 Naples, Italy; antimo.moretti@unicampania.it; 2 Department of Clinical Sciences and Translational Medicine, Faculty of Medicine and Surgery, “Tor Vergata” University of Rome, Via Montpellier 1, 00133 Rome, Italy; umberto.tarantino@uniroma2.it

The etymology of the word “athlete” derives from the ancient Greek ἀθλητής (athletés, from âthlos that is, fight, competition). The athlete, therefore, is the one who is striving in the effort to overcome a sport challenge, but, even more, in the effort to overcome himself. Sport-related musculoskeletal (MSK) disorders very commonly lead to poor performance and/or loss of competitions [1]. Several types and locations of MSK disorders might occur in athletes according to different sports, particularly affecting joints, skeletal muscles, and tendons. Considering the high frequency of these conditions and their significant impact on sport performance, several studies are focused on investigating their pathogenic mechanisms, as well as potential therapeutic approaches.

Although primary prevention mainly involves coaches and trainers, secondary and tertiary prevention of sport-related injuries, that are the reduction of reinjury, as well as their consequences to allow an effective sport participation, are the main challenges for healthcare professionals that treat athletes [2]. In this context, several unmet needs are required to be addressed, such as heterogeneous epidemiological data reporting of sport related MSK injury, natural history of the healing process of injured tissues, and safe return to play timing. Other topics of huge interest for physicians could be how different athletes respond to a specific injury and its complications, which outcomes are to be collected and monitored (e.g., return to play, physical function, pain, psychological, and/or socioeconomic measures) for assessing efficacy and/or effectiveness of different interventions, which management strategy or combination of treatments can modulate pathogenic mechanisms or address specific risk factors to reduce recurrence and/or to prevent disabling consequences of sport-related injury [2]. These conceptual and practical considerations should be investigated at both research (basic science and randomized controlled trials) and clinical levels (real-life practice), along with the athlete’s compliance, as well as the professional club agreement with proposed therapeutic strategies, and how medical specialists can implement these strategies. In this complex scenario, the Special Issue “Challenges and solutions for musculoskeletal disorders in athletes” aims to provide reliable and evidence-based answers to these challenging unmet needs.

In our opinion, the above mentioned issues can be addressed through a comprehensive and interdisciplinary teamwork that includes specialists with specific expertise in the management of MSK disorders, such as orthopedists, rheumatologists, and physiatrists, as well as by applying knowledge translation to stakeholders (e.g., clinicians) regarding debated sport-injury issues, such as diagnostic imaging pitfalls in muscle injury classification or the controversial role of exercise and other conservative measures in the occurrence and management of early osteoarthritis (EOA). This approach might overcome conceptual and language barriers among different stakeholders, and it can be better than simply adding each individual contribution in terms of decision-making and evidence-based interventions, finally contributing to the general wellbeing and safe and effective participation of athletes to sport practice.

The Special Issue has been endorsed by the Italian Society for Unified and Interdisciplinary Management of Musculoskeletal Pain and Algodystrophy (Società Italiana per la Gestione Unificata e Interdisciplinare del Dolore muscolo-scheletrico e dell’Algodistrofia, SI-GUIDA) to provide an overview of current epidemiological data, diagnostic challenges, and rationale of therapeutic strategies in the field of sport-related MSK disorders from the point of view of the three specialists most involved in the treatment of MSK pain, including the orthopedist, the rheumatologist and the physiatrist.

In particular, the Special Issue consists of ten articles of wide interest for clinicians that manage MSK injuries in athletes, starting from the definition and epidemiology of these lesions [3] and an update about the role of ultrasound (US) imaging in muscle injuries [4]. These lesions frequently affect lower limb muscles, particularly in football players and track and field athletes, leading to long recovery times and risk of re-injury. Diagnostic US has several practical advantages in this context, such as fast evaluation, portability, good spatial resolution, and the ability to perform dynamic tests.

The Special Issue also includes two papers addressing challenging conditions in sport practitioners, such as the bone marrow edema (BME) [5] and the Complex Regional Pain Syndrome (CRPS) type I [6] that call the clinician to face several pitfalls thus requiring early diagnosis and appropriate management to avoid disabling consequences. Bone marrow edema is an umbrella term used to define the instrumental findings of low signal intensity on T1-weighted (T1W) magnetic resonance imaging (MRI) and intermediate or high signal intensity findings on T2-weighted (T2W) MRI [7]. This condition is often reported in stress-related bone injuries of both professional and amatorial athletes, although its clinical significance remains unclear. Complex regional pain syndrome type I is a rare, chronic condition characterized by disproportionate pain, usually affecting distal limbs, that may develop in athletes following traumatic or overuse injuries that require a troublesome approach in term of diagnostic issues and therapeutic decisions [8,9,10,11,12,13].

Furthermore, the Special Issue includes six articles addressing pharmacological and non-pharmacological approaches to treat sport related MSK disorders. These papers provide an overview of the drug therapy for acute pain following sport-related trauma [14], a key issue for training and competition participation; a review about conservative treatment of sport-related tendinopathies with local injections of hyaluronic acid [15], a biologically active molecule secreted by synovial cells of the tendon sheath that contribute to shock absorbing and regenerative properties of tendons [16]; a focus on the biological role of vitamin D and clinical implications of its administration in skeletal muscle regeneration and function in athletes [17]. This latter is a topic of growing interest, considering that vitamin D modulates several functions of skeletal muscles, including tissue repair after injury, through vitamin D receptor expressed in satellite cells, and that vitamin D deficiency, reported also in athletes, results in fast-twitch fiber atrophy, fatty infiltration, and fibrosis. Finally, the Special Issue addresses commonly used non-pharmacological interventions for sport related MSK injuries, including therapeutic exercise and physical agent modalities for the management of EOA [18,19,20]. The development of EOA is a cause of concern for all people involved in sport practice, even if many open questions remain about its definition, natural history, and diagnostic criteria with relevant issues also in term of treatment strategies and timing of interventions [21]. Although highly demanding sports are considered risk factors for joint degeneration because of overload, repetitive microtrauma or other direct and indirect joint injuries, regular and progressive loading seems to positively affect articular cartilage. Indeed, exercise is a core treatment for EOA by increasing muscle strength, reducing fat mass, and enhancing viscoelastic properties of joint tissues thus preventing joint degeneration. Moreover, several physical modalities are commonly used as complementary treatment for patients with EOA, considering that physical agents trigger biological responses by regulating different intracellular pathways, thus acting as a drug.

In conclusion, the papers included in the Special Issue provide a comprehensive update of much debated topics in the field of MSK pathology focusing on the health issues that can affect and torment the athlete, putting at risk not only the early return to play, but also the professional career itself. The topics addressed from the point of view of MSK disorders specialists provide useful and practical information for both clinicians and researchers, also considering the unmet needs in the field of sport related MSK injuries.

## Data Availability

Not applicable.
